# An Opinion Interactive Model Based on Individual Persuasiveness

**DOI:** 10.1155/2015/345160

**Published:** 2015-10-05

**Authors:** Xin Zhou, Bin Chen, Liang Liu, Liang Ma, Xiaogang Qiu

**Affiliations:** School of Information System and Management, National University of Defense Technology, Changsha 410073, China

## Abstract

In order to study the formation process of group opinion in real life, we put forward a new opinion interactive model based on Deffuant model and its improved models in this paper because current models of opinion dynamics lack considering individual persuasiveness. Our model has following advantages: firstly persuasiveness is added to individual's attributes reflecting the importance of persuasiveness, which means that all the individuals are different from others; secondly probability is introduced in the course of interaction which simulates the uncertainty of interaction. In Monte Carlo simulation experiments, sensitivity analysis including the influence of randomness, initial persuasiveness distribution, and number of individuals is studied at first; what comes next is that the range of common opinion based on the initial persuasiveness distribution can be predicted. Simulation experiment results show that when the initial values of agents are fixed, no matter how many times independently replicated experiments, the common opinion will converge at a certain point; however the number of iterations will not always be the same; the range of common opinion can be predicted when initial distribution of opinion and persuasiveness are given. As a result, this model can reflect and interpret some phenomena of opinion interaction in realistic society.

## 1. Introduction

In the last decades, there are fruitful research achievements in learning behaviors of individual and complex group phenomena. There is little doubt that the behavior of individual and group phenomenon is inextricably linked. Group consists of lots of individuals, while common behaviors of plenty of individuals constitute many macroscopic emergences. Hence, it is an effective way to study the group phenomenon based on individual's behavior. Since the human society can also be considered as a complex multicomponent system consisting of individuals interacting with themselves and with their material environment, it is a challenge to develop a strategy allowing of a general quantitative modeling procedure for collective dynamic macro processes in the society [1]. In social dynamic, there is a premise that in social phenomena the basic constituents are not particles but humans and every individual interact with a limited number of peers, usually negligible compared to the total number of people in the system. So many macroscopic phenomena naturally call for a statistical physics approach to social behavior, which means the attempt to understand regularities at large scale as collective effects of the interaction among single individuals, considered as relatively simple entities [2].

In the research of cognitive learning behavior of individual, Brenner [3] sums up individual's cognitive behaviors as nonconscious learning [4, 5], routine-based learning [6], and belief learning [7, 8] according to the strength of the individual consciousness. However, in the research of complex emergence phenomenon, many researchers have put forward a number of models according to social dynamic, which derives from statistic physics, such as opinion evolution [9, 10], cultural dissemination [11], disease transmission [12, 13], and the spread of rumor [14, 15]. Among them, opinion dynamics has been a hot research field, which reflects and interprets a wide range of social phenomena ranging from collective decision making, finding and not finding of consensus, emergence of political parties, minority opinion survival, emergence of extremism, and so on [16]. In this paper, an opinion emergence phenomenon is studied based on opinion dynamics.

So far, models of opinion dynamics provided by scholars can depict the opinion interactive situation well in realistic society in some special situation. Based on the form of opinion, opinion dynamics can be divided into discrete model and continuous model. In discrete model, opinion is a discrete numerical variable which includes Voter Model [17], Sznajd Model [18], and Galam Model [19]. The idea of Voter Model is that an agent may be influenced by a neighbor so as to change its voting choice or opinion to the neighbor's and such local influences give rise to a global process of the collective voting results of the whole population. Sznajd Model states that one is easier to be persuaded by two or more people who sharing the same opinion than by a single person. Galam Model depicts that group consent is formed by the process of minority subordinating to the majority. In continuous model, opinion is a continuous variable which includes Deffuant model [20] and HK Model [21] mainly. Common places of these two models are that two individuals will not exchange their opinions until the difference of their opinions is below a threshold. However, in Deffuant model individual can only interact with one individual in each step while it can interact with many individuals in HK Model. Besides, there are a lot of expended models based on these two basic models.

In the early research, individual is homogeneous. With the deepening of researching, many scholars add the heterogeneous attribute to individuals, such as softhead individuals, amiable individuals, bigoted individuals, stubborn individuals, opinion leaders, and authoritative individuals [22]. In the past, the major networks of group are one-dimensional ring, grid, regular network, and fully connected network [22]. However, complex network, especially the discovery of small world network and scale-free network, injects new life into the research of opinion dynamics. The opinion dynamics in complex network [23, 24] and coevolution of opinion in self-adaptive network also become a hot topic gradually [25, 26].

This paper puts forward a new opinion interactive model based on Deffuant models and its improved models. This model emphasizes the importance of individual's persuasiveness and simulates the variation trend of group opinion. With this aim, the remainder of this paper is organized as follows. Previous work about Deffuant model is introduced in Section 2. A new model is then given in Section 3. After that, through simulation experiments, three problems in Section 4 are solved: the influence of randomness, initial persuasiveness distribution of group, and number of individuals on the common opinion, the influence of randomness, initial persuasiveness distribution of group, and number of individuals on the iteration of experiment, and the prediction of the interval of opinion common based on the initial distribution persuasiveness of group. Finally, in Section 5, we have a discussion about implication of our model.

## 2. Previous Works on Deffuant Model

The main idea of Deffuant model [20] is that, considering a population of *N* agents with continuous opinions *O*, at each time step any two agents are randomly chosen to meet. They readjust their opinion when their difference of opinion is smaller in magnitude than a threshold *d*. Suppose that the two agents have opinion *O*_*i*_ and *O*_*j*_ and that |*O*_*i*_ − *O*_*j*_ | < *d*, *i* and *j* represent the *i*th and the *j*th individual, respectively; opinions are then adjusted according to

(1) OiOi+μ·Oj−Oi,Oj=Oj+μ·Oi−Oj,

where *μ* is the convergent parameter taken between 0 and 0.5 during the simulations. The rationale for the threshold condition is that agents only interact when their opinion are already close enough; otherwise they do not even bother to discuss.

Honestly speaking, there are plenty of models after proposing of Deffuant model, especially some heterogeneous models. Lorenz [27] studies heterogeneous bounds of confidence, where two kinds of individuals, namely, open-minded and closed-minded individuals, are studied in the paper. The difference between open-minded and closed-minded individual is that they possess different *d*. In addition, extremism individual is studied by Weisbuch et al. [28]. The extremism model is based on two more assumptions: a few extremists with extreme opinions at the ends of the opinion spectrum and with very low threshold for interaction are introduced; whenever the threshold allows interaction, both opinions and threshold are readjusted according to similar expressions. That means in extremism model, the threshold *d* will change with the interaction; in other words, the more “tolerant” agent (with larger *d*) can be influenced by the less tolerant (with smaller *d*) while the less tolerant agent is not. What is more, truth seekers are discussed by Hegselmann and Krause [29] and Malarz [30]. In true seekers model, two parameters are added into Deffuant model: *T* ∈ [0,1] and *α*_*i*_ which represent the true opinion and the strength of the attraction to the truth for *i*th agent, respectively.

However, all the improved Deffuant models can only deal with one aspect problem. Most of them focus on the threshold *d*, while they seldom take the parameter *μ* into consideration. What is more, most of them divided group into several categories, like open-minded individual and close-minded individual. Nonetheless, it is true in realistic society that all the individuals are different from others. So, this paper analyzes a case that different individuals own different *μ*, namely, persuasiveness.

## 3. Model

Suppose a scene that a group of people discuss a topic. Everyone has its own attitude and interest to a certain topic. However people cannot only insist on their own opinion, because as an individual of society, it should take other people's opinion into account. After discussion, the group should reach an agreement. This scene often appears in reality society, such as the discussion of some topics in Congress and the discussion of entertainment place where to go in the weekend among office members.

This paper simulates the scene through modeling and experiments. Any two individuals chosen randomly in group can exchange their opinions. Opinions are published according to the round, and opinions of current round are only affected by opinions of last round. In the process of interaction, the sequence of speech of individual is ignored which means that the speech of the whole group is parallel. Individual acquires the last round opinion of itself and the opposite individual's opinion and then calculates the current round opinion of itself in line with some behavior rules and after that publishes its new opinion in the next round. With the evolution of group opinion, it forms a series of polymerization of macroopinion cluster eventually.

In this paper, the model is introduced in the form of agent. Individuals are abstracted as agents while each group consists of a lot of agents. Every agent has the same attributes and behavior rules, while they may be different from concrete attribute values or concrete behaviors. Through the interaction of microscopic interactive process, some macroscopic emergence phenomena of group can be found.

### 3.1. Attributes of Agent

Attributes of agent are abstracted as index, opinion, and persuasiveness. Explanation to these attributes is as follows.

#### 3.1.1. Index

To distinguish from other agents, every agent has a unique identity.

#### 3.1.2. Opinion

To a certain topic in the discussion, every agent has its own opinion. Opinion is the position or attitude that one observes things. In the mathematic model, opinion is abstracted as a discrete variable or continuous variable. Although it is maybe too simple to model the complex human society, it has some advantages to some certain problems, such as “turning left or turning right,” “approve or disapprove,” and “going to classroom or going to library.” Due to the variety of opinions to a certain topic and for the convenience of analysis, the opinion is modeled by continuous interval between 0 to 1 referring to Deffuant model. Among it, 0 and 1 represent the opposite opinion and 0.5 represents neutrality that opinion values have different meanings in different cases.

#### 3.1.3. Persuasiveness

Persuasiveness is the ability to persuade the other individual. In order to protect its own interest, everyone wants to persuade others and make them accept its opinion in the discussion. However, the status is often unequal. Some people are of higher qualification, elder age, or an opinion leader in some fields. So their opinions have a guiding role to others and their persuasiveness is higher than others. Most people have similar knowledge to a certain topic, and they do not have a deep research in it. So their opinions are just reference to others and their persuasiveness is lower. Drawing lessons from the mathematic model of opinion, the persuasiveness is modeled by continuous interval ranging from 0 to 1. Among it, 0 represents the lowest persuasion, 1 represents the highest persuasion, and 0.5 represents that individual has their own opinions to a topic but does not have an insight into it.

### 3.2. The Opinion Updating Rule of Agent

The paper does not take topology of network into consideration in this model. What we focus on is the influence of individual's persuasiveness and interactive probability on the process of interaction. In real life, if the difference between two individuals is low, the probability of thorough interaction between them may be high. However, it cannot be guaranteed that two sides will interact, because there are many other factors influencing interacting such as personal character. If the difference between two sides is large, the probability of thorough interaction is small. Similar to the former, it cannot guarantee that two sides will not interact of which the probability of interaction is just low. As to persuasiveness, an individual with high persuasiveness can change other's opinion easier than that with low persuasiveness.

Suppose that there are *N* individuals in finite set *A* = {1,2,…, *N*}, forming *N* × 1 opinion column vector {*O*_1_(*t*), *O*_2_(*t*),…, *O*_*N*_(*t*)}, where *O*_*i*_(*t*) is the *i*th individual's opinion in the *t* round, *O*_*i*_(*t*)∈[0,1], *i* ∈ *A*, *t* ≥ 0, where *t* = 0 is the initial opinion. Similarly, there are *N* × 1 persuasiveness column vector {*P*_1_, *P*_2_,…, *P*_*N*_}, where *P*_*i*_ is the *i*th individual's persuasiveness, *P*_*i*_ ∈ [0,1], *i* ∈ *A*. In our model, the persuasiveness of each individual is fixed and will not change with the time. For the convenience of description, the agent whose index is *i* is used to be the object of study, which is denoted as Agent *i*. With the above ideas, the opinion updating rules of Agent *i* are described as follows:

(1) each time Agent *i* randomly chooses an agent as partner, denoting the agent as Agent *j*;
(2) if (*p* < (1−|*O*_*i*_(*t*) − *O*_*j*_(*t*)|)), then enter into step (3); else exiting;
(3) (2) $$\begin{array}{c} O_{i}\left(\;t+1\;\right)=O_{i}\left(\;t\;\right)+P_{j}\cdot \left(\;O_{j}\left(\;t\;\right)-O_{i}\left(\;t\;\right)\;\right). \end{array}$$

Among formula (2), *p* is the random probability which means the probability of interaction, *p* ∈ [0,1], and obeys uniform distribution.

**Figure 1:**
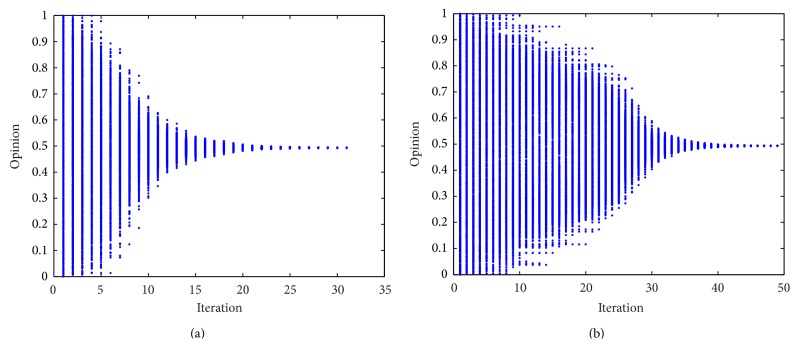
Classical opinion evolution processes (initial opinion distributions of group obey the uniform distribution ranging from 0 to 1); number of individuals is 1000. The horizontal ordinate represents the iteration number, and vertical ordinate represents individual opinion. (a) *p* = 0.5, *P*_*i*_ = 0.3; (b) *p* = 0.3, *P*_*i*_ = 0.3,  *i* ∈ *A*.

This model draws the lessons from Deffuant model and some other homogeneous Deffaunt models. Honestly speaking, if every individual possess the same and fixed parameters *p* and *P*_*i*_, there are no difference between our model and Deffuant model. However, when these two parameters are difference from person to person, many new issues should be solved.  Figure 1 shows the classical opinion evolution processes of Deffuant model.

From Figure 1 we can know that, with the increasing of the iteration, the group opinion gradually converges, forming the stable opinion cluster and reaching an agreement. Because the relationship network of the group is full connected graph, so the result of the model is influenced by randomness, initial opinion distribution, and persuasiveness of individual.

## 4. Simulation

This serial of experiments set a certain scale of agents, which does not take the topology of network into consideration. When the absolute difference of two opinions is less than 0.001, we assume that their opinions are the same. The simulation will come to an end when all the agents have the same opinion. The experiment flow is as follows:

(1) assign initial values to all the agents;
(2) in each time step, every agent can randomly choose one agent to exchange opinion. If the total number of individuals is odd, an agent will miss a turn in this round of interaction that it will not exchange its opinion with others;
(3) all the agents change their opinions based on the opinion updating rule in section three except the one who misses a turn;
(4) repeating step (2) and step (3), and if the whole group reaches an agreement, the simulation experiment stops.

In addition, for the convenience of analysis of the evolution character in macroscopic aspect, two indexes are defined here. Number of iterations (NOI) is the total time steps when all the agents have the same opinion, which indicates the convergent speed of group opinion. Common opinion (CO) is the opinion of group when all the agents have the same opinion, which determines variation trend of group opinion.

In the opinion updating model, there are two factors influencing CO and NOI. One factor is initial distribution of persuasiveness and opinion and the corresponding relationship between them. The other factor is the probability in the interaction. It is liable that the group may obey different kinds of distributions. However, for the convenience of analysis, we assume three persuasiveness distributions, namely, normal distribution, exponent distribution, and uniform distribution. Three experiments have been done in this paper. Experiment 4.1 and Experiment 4.2 are based on the same initial condition. The difference between them is that Experiment 4.1 focuses on the analysis of factors (influence of randomness, initial distribution, and number of individuals) influencing CO, while Experiment 4.2 stresses factors (influence of randomness, initial distribution, and number of individuals) influencing the NOI. Experiment 4.3 takes two initial distributions (normal distribution and exponential distribution) into account to predict the range of CO.

### 4.1. Influence of Randomness, Initial Distribution, and Number of Individuals on CO

In this experiment, we discuss three group experiments and each group experiment is also divided into three experiments. These three group experiments have the same initial opinion distribution obeying uniform distribution. The difference among them is that initial persuasiveness distribution of the first group experiment follows normal distribution, which can be denoted as “Nor,” initial persuasiveness distribution of the second group experiment follows uniform distribution which is denoted as “Unif,” and the remained group experiment follows the exponent distribution which can be represented as “Exp.” Each group experiment is also divided into three experiments, which are distinguished by the number of individuals. Each group experiment, respectively, has 100 agents, 1000 agents, and 10000 agents. So there are nine experiments in total; three experiments of “Nor” can be denoted as “Nor100,” “Nor1000,” and “Nor10000,” respectively; three experiments of “Unif” are represented as “Unif100,” “Unif1000,” and “Unif10000,” respectively; three experiments of “Exp” are denoted as “Exp100,” “Exp1000,” and “Exp10000,” respectively. Each experiment is independently repeatedly calculated for 100 times.

Random number generator separately generates initial opinion distribution and initial persuasiveness distribution of group based on above requirements. Suppose that the initial opinion distribution of all the experiments obeys the uniform distribution ranging from 0 to 1. As for “Nor100,” “Nor1000,” and “Nor10000,” the initial persuasiveness of group follows normal distribution of which the mean value *μ* is 0.5 and standard deviation *σ* is 0.1667. With regard to “Exp100,” “Exp1000,” and “Exp10000,” the initial persuasiveness of group obeys exponent distribution that the parameter *λ* is equal to 5. As to “Unif100,” “Unif1000,” and “Unif10000,” the initial persuasiveness of group follows uniform distribution of which the low bound is 0 and high bound is 1. If the generated persuasiveness is bigger than 0.99 or smaller than 0.01, it will be generated again. Here an extreme situation is taken into consideration; that initial opinion distribution and initial persuasiveness distribution are one-to-one correspondence from small to large in all these nine experiments, which can be labeled as positive sequence correspondence. Reasons for considering the situation is that on one hand it reflects some phenomena in real life, such as one proposal satisfies the desire of high persuasive people while it is not in line with the interest of low persuasive people; on the other hand it can deduce general situations.

Mean values and standard deviations of COs of nine experiments are shown in Table 1.

Based on Table 1, three phenomena can be found. (1) From each experiment, all the independently repeated experiments of “Nor100” converge at 0.5818; all the independently repeated experiments of “Nor1000” converge at 0.5978; other experiments similar to “Nor100” and “Nor1000” converge at different fixed values. (2) Classifying experiments based on the distribution, no matter how many agents they are, COs of “Nor100,” “Nor1000,” and “Nor10000” are all around 0.58; COs of “Unif100,” “Unif1000,” and “Unif10000” are all around 0.66; COs of “Exp100,” “Exp1000,” and “Exp10000” are all around 0.74. (3) Classifying experiments according to number of individuals, the CO of “Unif” is about 0.8 larger than “Nor” and that of “Exp” is about 0.8 larger than “Unif” when number of individuals is the same.

Without formula derivation, some conclusions can be drawn through these experiment results. (1) CO will converge at a fixed point when all the initial values of agents are fixed. From the perspective of experiment results, all COs of one group experiment are the same. From the perspective of mathematic, the order of magnitude of standard deviation is almost near 1.0∗10^−5^ in each group experiment in Table 1. The experiment precondition has supposed that two agent will reach an agreement when the difference of their opinions is less than 1.0∗10^−3^. The deviation can only influence the opinion value at 10^−4^ based on 3*σ* principle, so it will not change the opinion value at 10^−3^. In this way, the opinion will converge at a fixed point. (2) If initial persuasiveness of group obeys the same distribution and parameter, the number of individuals has little influence on CO. (3) The initial persuasiveness distribution has a big impact on CO, and different initial persuasiveness distributions leads to different COs. (4) Randomness has no effect on CO. According to the opinion updating rule, there are two random processes in the interaction, one process is two agents are randomly chosen to interact, and the other one is whether exchanging their opinions or not is based on probability. However, all the COs of 100 independently repeated experiments are the same in each group experiment. So it makes no difference to the CO based on experiment results.

### 4.2. Influence of Randomness, Initial Distribution, and Number of Individuals on NOI

**Figure 2:**
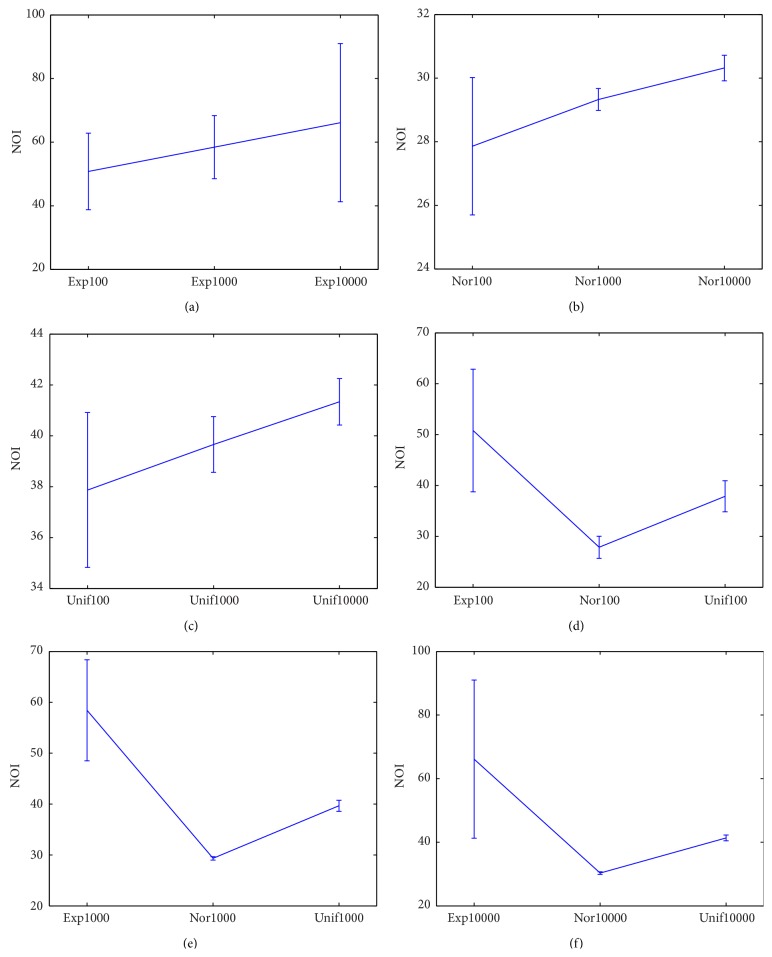
NOIs of nine experiments.

The initial condition of this experiment is the same with Experiment 4.1. In this experiment, the influence of randomness, initial distribution of agent's attributes, and the number of individuals on NOI are discussed.  Figure 2 shows the NOI of nine experiments. Among it, Figures 2(a), 2(b), and 2(c) show the difference of NOI when they have the same initial distribution but different numbers of individuals; Figures 2(d), 2(e), and 2(f) show the difference of NOI when they have the same number of individuals but different initial distributions. In these figures, horizontal coordinate represents different experiments, and vertical coordinate represents the mean value and deviation of NOI.

Many phenomena can be found from Figure 2. (1) From Figures 2(a), 2(b), and 2(c), the mean value of NOI of three distributions increases with the rising of number of individuals. Among them, the rising speed of mean value of “Exp” is the most obvious, where the mean value of “Exp10000” is about 15 larger than “Exp100”; the second is “Unif” where the mean value of “Unif10000” is about 4 larger than “Unif100”; and the least is “Nor” where the mean value of “Nor10000” is about 2 larger than “Nor100.” (2) From Figures 2(d), 2(e), and 2(f), the mean value of NOI of “Exp” is largest, and that of “Nor” is smallest when in the same number of individuals.

Without formula derivation, three conclusions can be acquired based on simulation experiment results. (1) Randomness is the basic cause of differences of NOI. The NOI is the same without randomness in the condition of the same initial value. (2) The more the initial distribution of persuasiveness is concentrated around 0.5, the less the NOI will be. From formula (1) we can deduce that it just needs one interaction that two sides of the interaction reach an agreement when their persuasiveness is 0.5. If their persuasiveness is far away from 0.5, it needs more than once to come to an agreement. Experiment results demonstrate it. In “Exp100,” “Exp1000,” and “Exp10000,” the initial persuasiveness distribution obeys exponent distribution of which the number of low persuasiveness individuals is large and the number of high persuasiveness individuals is small. In “Nor100,” “Nor1000,” and “Nor10000,” initial persuasiveness distribution follows normal distribution of which mean value is 0.5 and deviation is 0.1667. The NOI of “Exp” is much higher than that of “Nor” in the same number of agents. (3) With the increasing of number of agents, the NOI increases gradually. However, the speed of increasing depends on the initial persuasiveness distribution.

### 4.3. Two Predicted Experiments

In this experiment, a situation is considered that initial opinion distribution and initial persuasiveness distribution of group are known but we do not know the corresponding relationship between them. The CO cannot be acquired because the value of opinion and persuasiveness of every agent cannot be acquired in advance. Calculating the boundary condition is a proper solution which can decrease the degree of error of predicting CO. There are two situations of discussion in real life: one scene is that to a topic most people have their own opinion while only a few people have in-depth knowledge or have no idea of it; the other scene is that most people do not have idea of it while only a few people have an insight into the topic. Normal distribution and exponent distribution can substitute for these two scenes. Through simulation experiment, the range of CO in the light of initial persuasiveness distribution and initial opinion distribution can be predicted.

#### 4.3.1. Normal Distribution

The range of CO is based on two boundaries: one boundary is corresponding of positive sequence between initial persuasiveness distribution and initial opinion distribution, and the other boundary is corresponding of negative sequence between them. Opinion distribution and persuasiveness distribution are one-to-one correspondence from small to large in positive sequence corresponding while they are one-to-one correspondence that one distribution is from small to large and the other distribution is from large to small in negative sequence corresponding.

**Figure 3:**
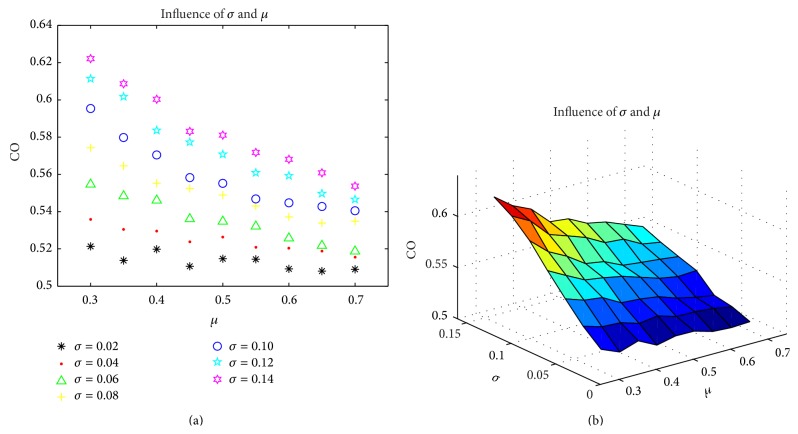
The influence of *μ* and *σ* on CO.

Positive sequence corresponding is taken into account at first. Suppose that the initial persuasiveness of group obeys normal distribution and initial opinion of group obeys the uniform distribution ranging from 0 to 1. The number of individuals is 10000. Because the range of persuasiveness is from 0 to 1, in order to confine the persuasiveness within the boundary it will generate persuasiveness again if persuasiveness is bigger than 0.99 or smaller than 0.01. The normal distribution has two parameters (deviation and mean value). A series of discrete values of standard deviation which are 0.02, 0.04, 0.06, 0.08, 0.1, 0.12, and 0.14 separately and discrete values of mean value which are 0.3, 0.35, 0.4, 0.45, 0.5, 0.55, 0.6, 0.65, and 0.7 separately are picked up.  Figure 3 shows experiment results. In Figure 3(a), horizontal ordinate represents the mean value, vertical ordinate represents the CO, and a series of different colors and shapes represent different standard deviations. In Figure 3(b), “miu” is the mean value, “sigma” is standard deviation, and CO is common opinion of group.

From Figure 3, the relationship among CO, *μ*, and parameter *σ* is estimated to obey a distribution which may be similar to two-dimensional normal distribution. Based on two-dimensional normal distribution, a proximate parameter equation is given as formula (3). The formula is one of the most suitable curves according to the simulation data and there are maybe other curves suiting the simulation data:

(3) $$\begin{array}{c} f\left(\;x,y\;\right)=a\cdot e^{\left(-b\cdot {\left(x-u\right)}^{2}-c\cdot {\left(y-v\right)}^{2}+d\cdot \left(x-u\right)\cdot \left(y-v\right)\right)}. \end{array}$$

Among it, parameters *a*, *b*, *c*, *d*, *u*, and *v* are real number ranging from negative infinity to positive negative. *x* represents mean value *μ*, *y* represents deviation *σ*, and *f*(*x*, *y*) is CO. After fitting the curve, the relationship among CO, *μ*, and *σ* is as formula (4). Consider the following:

(4) CO=0.5352∗e0.2804μ−1.0072−0.5445σ−0.13052−2.008μ−1.007σ−0.1305.

Table 2 shows four indexes of fitted curve. Sum of squared error (SSE) and root mean squared error (RMSE) are near 0; coefficient of determination (*R*-square) and degree-of-freedom adjusted coefficient of determination (adjusted *R*-square) are near 1. It means that the effect of fitting is well and unknown data can be predicted successfully.

**Figure 4:**
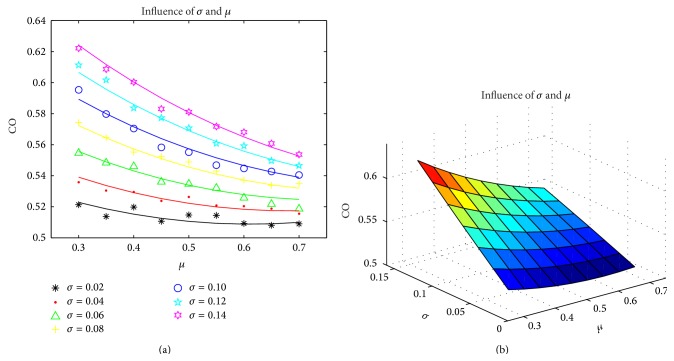
The fitted curve based on formula (4).

Figure 4 shows the result of fitted curve. Curves in Figures 4(a) and 4(b) are based on formula (4). Among them, the meaning of ordinates is the same with Figure 3.

In addition, the negative sequence corresponding is considered. All the conditions are the same with the above except the relationship of sequence corresponding between initial opinion distribution and initial persuasiveness distribution. However, it is not necessary to calculate again because the initial opinion distribution obeys uniform distribution ranging from 0 to 1 which has a symmetrical characteristic. CO of negative sequence corresponding is symmetrical with that of positive sequence corresponding and the axis of symmetry is *x* = 0.5, where *x* is the horizontal ordinate of initial opinion distribution.

Suppose that the initial persuasiveness distribution obeys normal distribution which mean that value is *μ*_0_ and standard deviation is *σ*_0_. Positive sequence corresponding is denoted as CO1 and the CO of negative sequence corresponding CO2. CO1 can be calculated from *f*(*μ*_0_, *σ*_0_), where *f*(·, ·) is formula (4) while CO2 = 1 − CO1. So the range of CO can be predicted approximately which is from CO1 to CO2.

#### 4.3.2. Exponent Distribution

**Figure 5:**
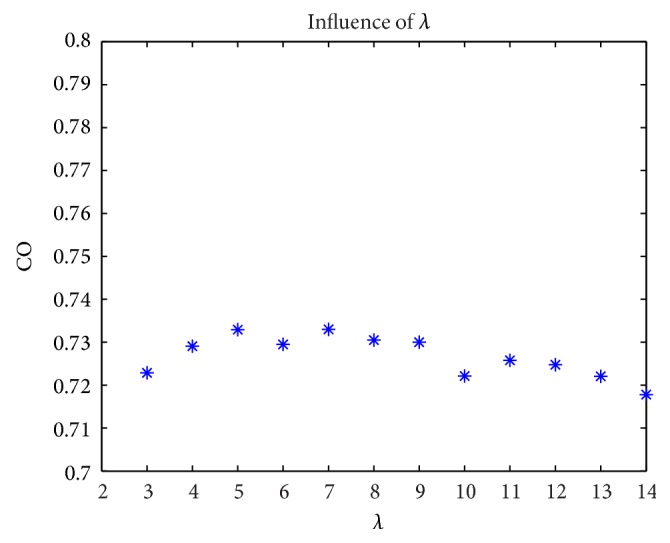
The influence of parameter *λ* on CO.

Positive sequence corresponding is taken into account at first. Suppose that the initial persuasiveness of group obeys exponent distribution and initial opinion of group obeys the uniform distribution ranging from 0 to 1. The number of individuals is 10000. Because the range of persuasiveness is from 0 to 1, in order to confirm persuasiveness within boundary, it will generate persuasiveness again if persuasiveness is larger than 0.99 or smaller than 0.01. The parameter in exponent distribution is denoted as *λ*. A series of discrete values of *λ* are set as 3, 4, 5, 6, 7, 8, 9, 10, 11, 12, 13, and 14 separately.  Figure 5 shows the experiment results. Horizontal coordinate represents parameter *λ*, while vertical coordinate represents the CO of group.

Figure 5 shows that parameter *λ* is nonsignificant where all COs are around 0.73. Hence, a conclusion can be acquired that the CO is equal to 0.73 approximately based on the premise that initial persuasiveness obeys exponent distribution and initial opinion obeys uniform distribution ranging from 0 to 1 which are positive sequence corresponding between them.

In addition, the negative sequence corresponding is considered. All the conditions are the same with the above except the relationship of sequence corresponding. However, it is not necessary to calculate again because the initial opinion distribution obeys uniform distribution ranging from 0 to 1 which has a symmetrical characteristic. The calculating process is the same with Experiment 4.3.1.

Suppose that the initial persuasiveness distribution obeys exponent distribution of which parameter is *λ*_0_. The result of positive sequence corresponding is denoted as CO1 and negative sequence corresponding as CO2. CO1 = 0.73 while CO2 = 1 − CO1 = 0.27. So range of CO can be predicted ranging from 0.27 to 0.73 approximately.

### 4.4. Conclusion

Through simulation experiments, some meaningful conclusions and predicted methods are summarized as follows.

(1) No matter how many independently replicate experiments we do, if initial values of all the individuals are fixed, the CO of group is fixed.
(2) Randomness, initial distribution, and number of agents have a great influence on the NOI of experiment. The more the initial persuasiveness distribution concentrates on 0.5, the less the NOI is. In the realistic society, the persuasiveness value of 0.5 represents that individual has their own opinions to a topic but does not have an insight into it.
(3) A method of predicting interval of CO is put forward in the condition that the distribution of initial opinion and initial persuasiveness are known in advance. When initial opinion distribution obeys uniform distribution ranging from 0 to 1 and initial persuasive distribution obeys normal distribution which mean that value is *μ*_0_ and standard deviation is *σ*_0_, the predicted interval of CO is from *f*(*μ*_0_, *σ*_0_) to 1 − *f*(*μ*_0_, *σ*_0_) where function *f*(·, ·) is formula (4). When initial opinion distribution obeys uniform distribution ranging from 0 to 1 and initial persuasive distribution obeys exponent distribution, the predicted interval of CO is from 0.27 to 0.73.

## 5. Discussion

The paper focuses on the influence of individual persuasiveness on opinion interaction and builds a new opinion interactive model based on Deffuant models and its improved models. The most different thing between our model and Deffuant model is that all individuals in our model are different from others, namely, owning different persuasiveness. If such a mechanism of taking a decision by a community is correct, our model leads to the following conclusions.

1. In a closed (isolated) community there are a variety of possible opinions ranging from person to person in original state. After a short and long time our model tend to be one of the “ordered” states, there is a possibility of reflecting the case that the group takes a common decision.
2. As for a certain discussion topic of closed community, when the initial conditions of the whole group are known, the result may be predicted ahead of time. What may not be predicted easily is the convergent time because of many uncertain factors, like number of individuals, individual persuasiveness, and so forth.
3. One of the most important factors is the distribution of persuasiveness. Taking Section 4.3.2, for example, the initial persuasiveness distribution of group follows the exponent distribution. Although the proportion of high persuasiveness individuals is much less than that of low persuasiveness individuals, the common opinion is always near the opinion of high persuasiveness individual. By what I mean is that the public (with low persuasiveness) may easily be influenced by opinion leader (with high persuasiveness) and change their opinions to approach the opinion leader which frequently occur in today's society; especially some people are unaware of the truth. So, in order to make the group approach one's opinion, the most crucial thing is to improve his persuasiveness, rather than the number of individuals.

To sum up, the proposed very simple rules trigger a rather complicated dynamics. However, one can doubt if these rules properly describe real mechanisms of taking a decision. There are of course other possibilities within the improved model. Because humans are exactly the opposite of such simple entities, such as atoms and molecules, indeed the detailed behavior of each of them is already the complex outcome of one's interest, benefits, and especially many physiological and psychological processes. However in the model, we just consider a certain case mentioned in Section 3. And these simulation results reflect and interpret some social phenomena of opinion interaction to an extent; for example, persuasiveness of individual is far more significant than the number of individuals in the discussion, which has an important guiding significance of predicting the common opinion of group.

## Figures and Tables

**Table 1:** COs of nine experiments.

Group name	Nor100	Nor1000	Nor10000
Mean value	0.5818	0.5978	0.5838
Deviation	7.7987∗10^−6^	1.5884∗10^−6^	2.7965∗10^−6^

Group name	Unif100	Unif1000	Unif10000
Mean value	0.6353	0.6643	0.6656
Deviation	1.1000∗10^−5^	2.5195∗10^−6^	4.9222∗10^−6^

Group name	Exp100	Exp1000	Exp10000
Mean value	0.7272	0.7482	0.7500
Deviation	1.1030∗10^−5^	3.0887∗10^−6^	5.6305∗10^−6^

**Table 2:** Indexes of fitted curve.

Index	Value
SSE	0.0005162
RMSE	0.003009
*R*-square	0.9896
Adjusted *R*-square	0.9887
